# Partial Lipodystrophy and LMNA p.R545H Variant

**DOI:** 10.3390/jcm10051142

**Published:** 2021-03-09

**Authors:** Silvia Magno, Giovanni Ceccarini, Andrea Barison, Iacopo Fabiani, Alessandro Giacomina, Donatella Gilio, Caterina Pelosini, Anna Rubegni, Michele Emdin, Gian Luca Gatti, Filippo Maria Santorelli, Maria Rita Sessa, Ferruccio Santini

**Affiliations:** 1 Obesity and Lipodystrophy Center, Endocrinology Unit, University Hospital of Pisa, 56124 Pisa, Italy; silviamagno9@gmail.com (S.M.); giovanni.ceccarini@unipi.it (G.C.); donatella.giliomd@gmail.com (D.G.); 2 Fondazione Toscana Gabriele Monasterio, 56124 Pisa, Italy; abarison@ftgm.it (A.B.); iacopofabiani@gmail.com (I.F.); m.emdin@santannapisa.it (M.E.); 3 Institute of Life Sciences, Scuola Superiore Sant’Anna, 56124 Pisa, Italy; 4 Plastic and Reconstructive Surgery Unit, Hospital of Pisa, 56124 Pisa, Italy; a.giacomina@gmail.com (A.G.); gianlucagatti@alice.it (G.L.G.); 5 Chemistry and Endocrinology Laboratory, University Hospital of Pisa, 56124 Pisa, Italy; caterina0376@hotmail.com (C.P.); m.sessa@ao-pisa.toscana.it (M.R.S.); 6 IRCCS Fondazione Stella Maris, 56100 Pisa, Italy; anna.rubegni@fsm.unipi.it (A.R.); filippo3364@gmail.com (F.M.S.)

**Keywords:** lipodystrophy, familial partial lipodystrophy type 2, FPLD2, leptin, *LMNA* mutation

## Abstract

Laminopathies are disorders caused by *LMNA* gene mutations, which selectively affect different tissues and organ systems, and present with heterogeneous clinical and pathological traits. The molecular mechanisms behind these clinical differences and tissue specificity have not been fully clarified. We herein examine the case of a patient carrying a heterozygous *LMNA* c.1634G>A (p.R545H) variant with a mild, transient myopathy, who was referred to our center for the suspicion of lipodystrophy. At physical examination, an abnormal distribution of subcutaneous fat was noticed, with fat accumulation in the anterior regions of the neck, resembling the fat distribution pattern of familial partial lipodystrophy type 2 (FPLD2). The R545H missense variant has been found at very low allelic frequency in public databases, and in silico analysis showed that this amino acid substitution is predicted to have a damaging role. Other patients carrying the heterozygous *LMNA* p.R545H allele have shown a marked clinical heterogeneity in terms of phenotypic body fat distribution and severity of organ system involvement. These findings indicate that the *LMNA* p.R545H heterozygous variant exhibits incomplete penetrance and highly variable expressivity. We hypothesized that additional genetic factors, epigenetic mechanisms, or environmental triggers might explain the variable expressivity of phenotypes among various patients.

## 1. Introduction

Mutations in the *LMNA* gene can result in a wide range of disease phenotypes, collectively termed laminopathies, which involve different tissues and organ systems causing cardiomyopathies (conduction-system diseases and dilated cardiomyopathy), myopathies (type 2 Emery-Dreifuss muscular dystrophy-EDMD2, type 1B limb-girdle muscular dystrophy-LGMD1B), neuropathies (Charcot–Marie–Tooth disease type 2B1- CMT2B1), lipodystrophies (most commonly type 2 familial partial lipodystrophy), premature aging syndromes (Hutchinson-Gilford progeria syndrome-HGPS, Type A mandibuloacral dysplasia-MADA, restrictive dermopathy-RD, atypical progeroid syndrome-APS), and overlap syndromes characterized by a combination of various phenotypes in the same subject, involving adipose tissue, skeletal/cardiac muscle, and/or the peripheral nervous system [1,2,3,4]. The causative molecular mechanisms behind this clinical heterogeneity and tissue specificity are still poorly understood [3,5].

The *LMNA* gene is located on human chromosome 1q21-22, and via alternative splicing, encodes 2 major protein isoforms: lamin A (full form, exons 1–12) and lamin C (short form, exon 1–10), which are components of the “nuclear lamina”, a network of proteins involved in nuclear structure, organization, and communication [6,7]. Lamins A and C consist of a short N-terminal head, a central α-helical rod domain, and a large C-terminal tail comprising a globular immunoglobulin-like domain (IgG-like domain). These proteins are expressed in a variety of differentiated tissues, and their structural integrity is critical to human health.

Hierarchical cluster association studies have shown that phenotypes characterized by primary cardiac and neurological involvement are more frequently associated with *LMNA* mutations located in the helical rod domain, upstream of the nuclear localization signal sequence (NLS), placed at codons 416–423, while diseases affecting primarily the adipose tissue or leading to premature ageing are likely caused by mutations located in the tail or immunoglobulin (IgG)-like domain, downstream of the NLS [8,9].

We herein report on the clinical history of a patient affected by an overlap syndrome caused by heterozygous *LMNA* c.1634G>A (p.R545H) variant in exon 10 and review the previously described cases.

## 2. Case Description

**Figure 1:**
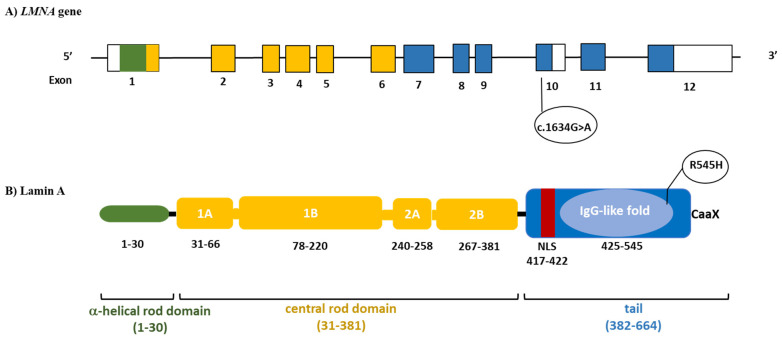
Schematic structure of *LMNA*. In the upper panel, filled boxes indicate the exons of the gene (**A**). Protein domains are displayed color-coded in the lower panel (**B**). The heterozygous *LMNA* variant detected is also reported. NLS, nuclear localisation signal.

The patient is a Caucasian girl, the only child of healthy non-consanguineous parents. She was born with a normal birth weight after an uneventful pregnancy. Her psychomotor development was reported as normal in the first 6 months after birth. However, between 8 and 9 months of age, some growth and motor delays became progressively evident: her head control became impaired and she was not able to maintain a sitting position. Brain magnetic resonance imaging (MRI) did not show abnormalities. At the age of 15 months, a muscle biopsy showed the presence of myopathic features with abnormal oxidative metabolism, raising the suspicion of a possible primary mitochondrial disease (MD). In the following months, myopathic symptoms improved, and the patient started walking between 18 and 20 months of age. Subsequent psychomotor development was normal, although the patient complained of mild muscle weakness. She had menarche at 11 years of age and thereafter continued to have regular menstrual cycles. At the age of 12 years, she developed progressive fat accumulation in the face and neck. An electromyography (EMG) of the upper extremities did not reveal pathological findings, while a thigh MRI showed areas of fat infiltration affecting the vastus lateralis muscle. A second muscle biopsy showed mild variation in fiber size, with some internal nuclei associated with unremarkable stain of oxidative metabolism enzymes and normal expression of the respiratory chain complex enzyme activities. Genetic testing revealed a heterozygous missense *LMNA* mutation c.1634G>A causing an amino acid change (p.R545H) in exon 10 (Figure 1). Because of the genetic and clinical suspicion of partial lipodystrophy, at the age of 17 years, the patient was referred to our attention.

**Figure 2:**
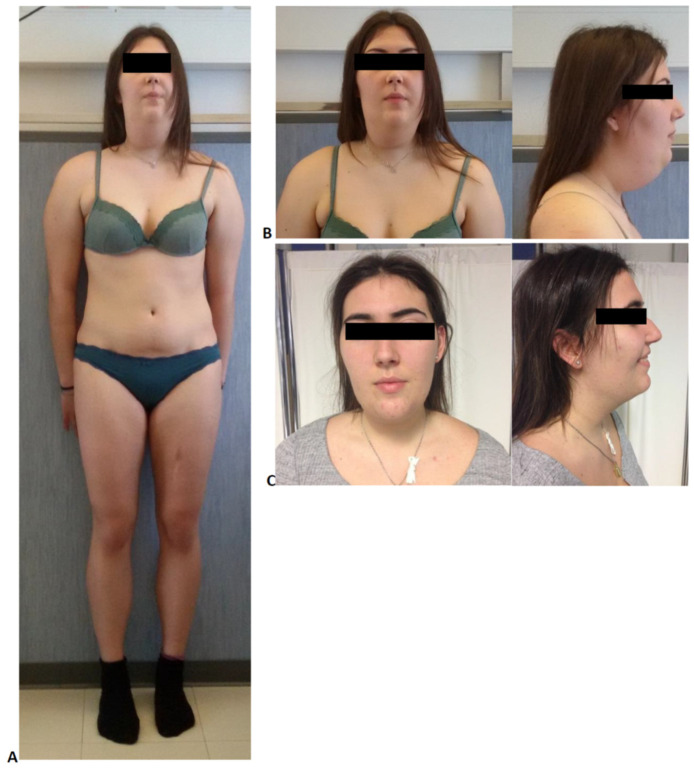
Pictures of the patient carrier of the heterozygous *LMNA* pR545H mutation. (**A**,**B**) Anterior and lateral view of the patient taken two years before (at age 17) plastic surgery intervention. Excess of fat over the shoulders and in the anterior regions of the neck along the jawline is evident without clear signs of lipoatrophy. (**C**) Anterior and lateral view of the patient taken one month after the plastic surgery intervention (liposuction).

At physical examination, an abnormal distribution of subcutaneous fat was noticed, with fat accumulation over the shoulders and in the anterior regions of the neck along the jawline, giving her the appearance of a double chin (Figure 2A,B). Body height and body mass index were normal: 1.72 m and 24 kg/m^2^, respectively (Table 1). She had no signs of hirsutism, virilization, or acanthosis nigricans. The last clinical neurological examination was within normal limits.

Biochemical blood tests (Table 2) showed normal blood glucose, insulin and glycated hemoglobin, triglycerides, cholesterol, and liver enzymes. The 2 h, 75 g oral glucose tolerance test was also normal. Creatine phosphokinase (CPK) levels were mildly elevated (390 U/L), although subsequent blood tests displayed normal values.

The endocrinological evaluation showed normal thyroid, gonadal, pituitary, and adrenal function. Plasma leptin and adiponectin levels were 10.4 ng/mL and 5 mcg/mL, respectively. Table 1 shows the results of skinfold thickness measurement (triceps, thigh, and calf) and the percentage of leg fat assessed by dual-energy X-ray absorptiometry (DXA): values were higher compared with those measured in a cohort of patients with typical FPLD2 but were at the lower limit of normal compared with unaffected controls [13,14].

**Figure 3:**
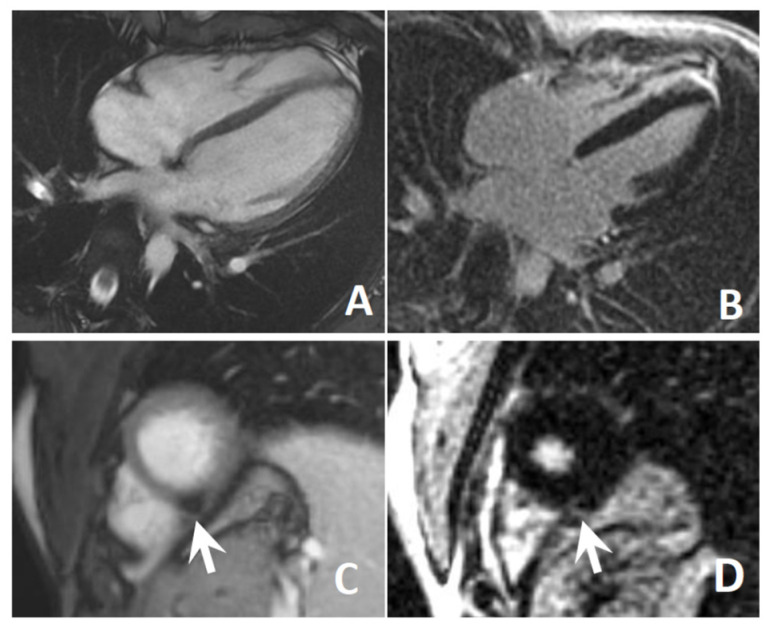
Top: Cardiac magnetic resonance four-chamber view showing normal biventricular volumes and wall thickness at cine steady-state free-precession (**A**) with no apparent areas of fibrosis at late gadolinium enhancement (**B**). Bottom: Short axis apical view showing a small, non-specific fibrofatty area in the distal interventricular septum appearing dark at cine steady-state free-precession sequences (**C**) and bright at late enhancement sequences (**D**).

Abdominal ultrasonography revealed a normal liver volume without signs of hepatic steatosis. Cardiological evaluation showed normal findings at standard 12-lead electrocardiogram (ECG), 24 h Holter ECG recording, and transthoracic echocardiography. In particular, the patient presented a normal biventricular systolic function (left ventricular ejection fraction 60% by 2D/3D echo, global longitudinal strain—18%), a preserved functional capacity in the absence of wall motion abnormalities during exercise, and a nearly normal rest ECG (sinus rhythm with mild repolarization abnormalities). Her biohumoral profile resulted within normal limits (high-sensitivity troponin T, hs-TnT 5 ng/L; N-terminal proB-type natriuretic peptide, NT-proBNP, 14 ng/L). Cardiac magnetic resonance (CMR) findings were also normal, except for a focal area of non-ischemic fatty infiltration in the distal interventricular septum (Figure 3). Because of her complaints regarding body image, at the age of 18 years, she underwent plastic surgery intervention (liposuction) for double-chin reduction (Figure 2C).

Her parents did not display phenotypic abnormalities suggestive of lipodystrophy (her mother is overweight, and her father has a normal weight). Genetic testing showed the presence of the missense heterozygous *LMNA* mutation c.1634G>A (p.R545H) in her father. His clinical assessment did not show any sign of lipoatrophy and/or fat overaccumulation, his neurological examination was normal, and his laboratory tests were all within normal levels (Table 1 and Table 2). No pathological findings were observed in abdominal ultrasonography or cardiologic evaluation (ECG and echocardiography). Muscle biopsy showed mild variation in fiber size for the presence of some atrophic fibers and increased internal nuclei.

## 3. Methods

### 3.1. Anthropometric Measurements

Height and body weight were measured by standard procedures. Skinfold thickness was measured with a Lange caliper (Beta Technology, Santa Cruz, CA, USA) at truncal (abdomen, subscapular, and suprailiac) and four peripherals (biceps, triceps, mid-thigh, and calf) sites on the right of the body. The average of three repeat measurements at each site was calculated.

Total and segmental body fat in the trunk, upper and lower extremities was evaluated by whole-body dual-energy X-ray absorptiometry (DXA, Hologic, Discovery A, S/N 84551). The proportion of fat in specific body districts as well as the whole body was calculated as a percentage of body mass.

### 3.2. Biochemistry and Hormones

All determinations were carried out after at least 12 h of fasting. Leptin and adiponectin were measured by ELISA from Mediagnost, Reutlingen, Germany. Hormones, glucose, cholesterol, triglycerides, creatinine, AST, ALT, and GGT were determined using automated equipment at the Chemistry and Endocrinology Laboratory at the University Hospital of Pisa, Italy.

### 3.3. Genetic Testing

*LMNA* variants were identified by Sanger sequencing and further confirmed on a second DNA sample. Specific primers were designed using Primer 3 (http://primer3.ut.ee/Primer3web version 4.1.0, last accessed on 8 March 2021) to amplify the coding exons and splice junctions from genomic DNA, isolated from whole blood. PCR was performed using PCR Master Mix (Promega Corporation, Madison, WI 53711-5399, USA), with an annealing temperature of 55 °C. After purification with ExoProStar (GE Healthcare UK Limited, Amersham, UK), the PCR products were directly sequenced using Applied Biosystems 3130 xl sequencer (Thermo Fisher Scientific, Waltham, MA, USA).

Variant frequency was investigated in the general population by accessing data from the 1000 Genomes project (https://www.internationalgenome.org/, accessed on 8 March 2021) and The Genome Aggregation Database (gnomAD) (https://gnomad.broadinstitute.org/, accessed on 8 March 2021). The *LMNA* p.R545H variant has been found at low allelic frequency in the 1000 Genomes Project (0.00020) and in the Genome Aggregation Database (0.0002474).

The impact of the detected missense variant on protein function was assessed as deleterious by four different in silico prediction algorithms (Table 3).

### 3.4. Cardiological Assessment

The patient underwent a cardiovascular work-up including rest and supine-bicycle exercise echocardiography; 12-lead resting ECG and 24 h Holter evaluation (Cardioline, Italy); biohumoral profile including hs-TnT; and NT-proBNPEchocardiographic evaluation including wall thickness, chamber volumes, and indices of systolic (including 2D and 3D ejection fraction, EF% and Global longitudinal strain, GLS %) and diastolic function assessment [15].

Cardiac magnetic resonance (1.5 T, Signa Artist, GE Healthcare, Waukesha, WI, USA) was performed to assess biventricular systolic function (steady-state free precession cine sequences) and late enhancement imaging (T1-weighted gradient-echo inversion-recovery sequence acquired 10–20 min after 0.2 mmol/Kg gadoteric acid injection) [16].

## 4. Discussion

Laminopathies are a heterogeneous group of inherited disorders caused by mutations in the *LMNA* gene. In some cases, these mutations may give rise to distinct clinical entities or may generate overlap syndromes with heterogenous phenotypes. In laminopathies there is no strict genotype–phenotype correspondence; similar diseases can therefore be the result of different mutations [17].

The case description is an intriguing example of an overlap syndrome caused by *LMNA* c. 1634G>A (R545H) mutation, characterized by fat overaccumulation in the shoulder and neck areas and mild, transient myopathy.

*LMNA* p.R545H causes the substitution of an arginine (a positive polar amino acid) with a histidine (a neutral polar amino acid) in exon 10, common to both lamins A and C that lie in the IgG-like domain. Mutations in the Ig-fold domain (residues 428–549) can interfere with lamin polymerization and interactions with other proteins [18]. This variant is present at very low allelic frequencies in public databases (1000 Genomes Project: 0.00020; The Genome Aggregation Database: 0.0002474).

We analyzed the pathogenicity score of the LMNA p.R545H substitution using in silico predictors. Available in silico tools can be classified into different categories: those based on the assumption that a disease-causing missense mutation is likely when a great difference between the physicochemical properties of substituted amino acids is present (sequence-based tools); and those based on the assumption that a disease-causing mutation is likely when located at a highly conserved site across species (structure-based tools) and those utilizing both criteria (protein structure/function and evolutionary conservation).

We performed our in silico analysis using four different prediction tools (Table 3), which took advantage of both protein structure/function relationship and evolutionary conservation, and showed a full concordance among them on variant pathogenicity. It is worth mentioning that the “pathogenic score” is an indicator of likelihood of pathogenicity and does not correlate with the phenotypic spectrum of the variant. The substitution of the arginine with a cysteine at this position (p.R545C), a highly conserved site among different species, has been described in a patient with a severe Emery-Dreifuss muscular dystrophy. Myoblasts in the culture from this patient showed defects of the organization of both lamin A/C and its partner emerin, confirming the critical role of the arginine residue in position 545 [19]. The same amino acid substitution at the nearby 541 position (R541H) has been associated with cardiomyopathy [20]. Taken together, these data indicate the importance of arginine at position 545 and support the pathogenicity of the detected missense variant. In the literature, only four cases with heterozygous *LMNA* p.R545H mutation have been described so far. Their clinical features are described in Table 1. Of particular note is the marked clinical heterogeneity in terms of lipodystrophic phenotypes and severity of the disease. Our patient showed an accumulation of adipose tissue over the shoulders and in the neck area, resembling the pattern of fat distribution of type 2 familial partial lipodystrophy (FPLD2), also known as Dunnigan Syndrome. The latter is the most frequent laminopathy associated with partial lipodystrophy, and it is usually caused by heterozygous variants in exon 8, especially the p.R482W/Q one, although variants in other exons have been described [21,22,23]. In our patient, similar to classic Dunnigan disease, the excess of subcutaneous fat in the neck (chin and supraclavicular region) became evident during puberty, but it was not associated with evident lipoatrophy of the four limbs and buttocks.

At variance with our patient, previously described carriers of the *LMNA* p.R545H variant displayed android fat distribution associated with mild fat loss in the lower limbs and inconsistent fat neck accumulation; furthermore, two out of four patients were affected by obesity [10,11,12].

In this patient, myopathy was the initial manifestation of the disease even if, at the time we first evaluated her, she presented with very mild muscle weakness and occasionally increased creatine kinase levels. In agreement with the foremost characteristics of the *LMNA*-related myopathy [24], EMG and muscle histological findings displayed an “unspecific” myopathic pattern, while muscle MRI revealed a fatty infiltration. Among the previously published cases, another patient presented with myopathy and high levels of creatine kinase. As this patient was affected by chronic hypercortisolism, it is therefore not possible to establish whether this condition had influenced the myopathy. However, once the hypercortisolism was cured, CK levels worsened, demonstrating persistent muscle impairment. Muscle involvement in *LMNA* c. 1634G>A (R545H) mutation has been previously reported in a patient with Emery-Dreifuss muscular dystrophy [25]. In this case, the patient’s mother harbored the same mutation but was completely asymptomatic, indicating the incomplete penetrance of this mutation.

R545H *LMNA* variant has been previously associated with cardiac abnormalities, although a detailed characterization of these patients is lacking [26,27]. The association between *LMNA* mutation, lipodystrophy, and cardiac involvement (heart conduction disorders, valvulopathies, and/or cardiomyopathies) has been amply reported, usually manifesting clinically after the third decade, with very high penetrance by the age of 70 years [26]. However, cardiac involvement cannot be predicted only on the basis of the specific *LMNA* mutation [28]. Our patient did not display any cardiac manifestations, with the exception of the area of myocardium fatty infiltration. However, in view of her young age, we cannot rule out the possibility that these may arise in the future. Among the patients affected by *LMNA* R545H variant described so far, one had atrial fibrillation associated with Wolff–Parkinson–White syndrome [10].

It is important to point out that most of the patients reported so far harbored the *LMNA* p.R545H variant in the heterozygous state, but recently, Patni et al. described a case of two sisters with a homozygous *LMNA* p.R545H mutation. They showed a more severe phenotype, with almost generalized fat loss and severe metabolic complications (diabetes mellitus, hepatic steatosis, and marked hypertriglyceridemia) associated with other complex features (early onset intellectual disability, short stature, clinodactyly). In contrast, their relatives with the monoallelic variant did not present any signs of lipodystrophy or any other disease associated with laminopathy [12].

Different *LMNA* variants have been described in their homozygous state; in these patients, severe disease was observed to be associated with almost generalized fat loss [23,29,30]. Interestingly, these different case descriptions converge on the evidence of incomplete penetrance and a highly variable disease expressivity in the heterozygous state, even within the same family [23,29,30].

In line with these observations, our patient’s father was an asymptomatic carrier of the p.R545H variant; he did not show any phenotypic abnormalities suggestive of lipodystrophy, metabolic alterations, or signs of clinically evident myopathy or cardiac involvement. This may also occur in type 2 familiar partial lipodystrophy (FPLD2), where male subjects do not always display the signs of the disease because they usually exhibit less pronounced subcutaneous fat loss and metabolic alterations compared to female subjects [14,31].

## 5. Conclusions

These observations suggest that the *LMNA* p.R545H variant displays an autosomal dominant transmission with incomplete penetrance and highly variable expressivity among affected individuals, even within the same family. Additional background genes with specific variants, as well as epigenetic mechanisms of gene regulation or environmental factors, might explain the variable expressivity of the disease.

Due to the heterogeneity of the *LMNA*-related lipodystrophy syndromes, affected patients may struggle before their disease is correctly diagnosed. We therefore emphasize that it is important to suspect the presence of *LMNA* mutation in patients with unusual subcutaneous fat distribution to allow for proper diagnosis of milder forms. In this regard, we believe that appropriate disease registries [32] will be able to improve diagnostics, patient care, and therapeutic choices.

## Figures and Tables

**Table 1:** Clinical features of our index case patient with heterozygous *LMNA* p.R545H variant compared with previously published cases.

	Index Case	Father of the Index Case	Patient 3	Patient 4	Patient 5	Patient 6	Patient 7	Patient 8
	Current Study	Current Study	[10]	[11]	[12]	[12]	[12]	[12]
**Sex**	F	M	F	F	F	M	F	F
***LMNA* pR545H**	HT	HT	HT	HT	HT	HT	HO	HO
**Age at report (y)**	21	45	48	51	40	16	16	16
**Height (m)**	1.72	1.74	1.61	1.62	1.55	1.64	1.40	1.44
**Weight (kg)**	71	72	65.3	61.1	96.8	100.2	32.2	37.8
**BMI (kg/m2)**	24	23.8	25.2	23.1	40.2	36.9	16.4	18.1
**Lipoatrophy**	-	-	Mild (limbs and buttocks)	Mild (limbs and buttocks)	-	-	Severe (Trunk limbs and buttocks)	Severe (Trunk limbs and buttocks)
**Fat Accumulation**	Neck	-	Face/Neck/Abdomen	Neck/Abdomen	Obese	Obese	-	-
**Acanthosis Nigricans**	-	-	NR	NR	+	+	-	NR
**Hepatic steatosis**	-	-	NR	+	NR	NR	+	+
**Hypertriglyceridemia**	-	-	+	+	-	-	+	+
**Diabetes/IR**	-		+	+	+	-	+	+
**Cardiovascular Involvement**	-	-	WPW syndrome	Hypertension	-	-	NR	NR
**Myopathy**	+	-	+	-	-	-	-	NR
**Additional characteristics**	-	-	-	-	-	Umblical hernia, orchidopexy for UDT	Intellectual disability, clinodactyly, joint contractures, cataracts, uterine leiomyoma	Intellectual disability, clinodactyly, joint contractures, cataracts, uterine leiomyoma
**Whole body fat (%)**	31.5	20.9	30.6	-	39.9	-	22.3	26.8
**Arm fat (%)**	43	17.9	31.6	-	55.9	-	25	27.6
**Leg fat (%)**	33.9	20.8	30.5	-	34.1	-	17	16.5
**Truncal fat (%)**	26.8	22.3	31.8	-	39.2	-	24.1	32.2
**Skinfold thickness**								
**Abdomen (mm)**	21	22	-	-	-	-	12	16
**Suprailiac (mm)**	9	15	15	-	-	-	10	12
**Subscapular (mm)**	15	16	18	-	-	-	7	11
**Biceps (mm)**	11	5	10	-	-	-	2	6
**Triceps (mm)**	18	12	17	-	-	-	4	4
**Midthigh (mm)**	16	13	10	-	-	-	6	5
**Calf (mm)**	10	7	5	-	-	-	3	6

Footnotes: HT, heterozygous; HO, homozygous; IR, insulin resistance; NR, not reported.

**Table 2:** Biochemical tests of the patients described.

	Index Case	Father of the Index Case	* Patient 3	Patient 4	Patient 5	Patient 6	Patient 7	Patient 8
	Current Study	Current Study	[10]	[11]	[12]	[12]	[12]	[12]
Glucose								
Fasting (mg/dL)	85	84	65–91	99	85	83	292	146
1 h OGTT (mg/dL)	136	139	-	NR	-	-	-	-
2 h OGTT (mg/dL)	94	102	-	229	-	-	-	-
HbA1c (%)	5	5.4	6–6.1	NR	6.1	5	7.2	6.7
Insulin								
Fasting (µUI/mL)	9.76	2.62	43.9	NR	NR	NR	NR	NR
1 h OGTT (µUI/mL)	70.1	40.7	-	NR	-	-	-	-
2 h OGTT(µUI/mL)	38.8	20.5	-	NR	-	-	-	-
Lipids								
Total-C (mg/dL)	161	176	63–139	319	208	165	>1000	210
HDL-C (mg/dL)	54	73	40–40	42	51	37	10	37
LDL-C (mg/dL)	100	113	NR	NR	185	109	-	-
TG (mg/dL)	65	54	202–214	230	185	99	5436	262
Liver function								
AST (U/L)	38	15	NR	NR	18	20	60	45
ALT (U/L)	25	17	NR	39	17	21	111	77
γGT(U/L)	10	13	NR	NR	NR	NR	NR	NR
CPK (U/L)	390-46-70	88	378–2500	50	NR	NR	NR	NR
LDH (U/L)	221	157	NR	NR	NR	NR	NR	NR
Leptin (ng/mL)	10.4	1.4	1–8.4	NR	NR	NR	1.9	9.1
Adiponectin (mcg/mL)	5	-	NR	NR	NR	NR	<2	2

Footnotes: ALT, alanine aminotransferase; AST, aspartate aminotransferase; CPK, creatine phosphokinase; γGT, gamma glutamyl transferase; HbA1c, hemoglobin A1c; HDL-C, high-density lipoprotein cholesterol; LDL-C, low-density lipoprotein cholesterol; 1 hOGTT, 1 h after oral glucose tolerance test; 2 hOGTT, 2 h after oral glucose tolerance test; Total-C, total cholesterol; TG, Triglycerides; NR, not reported * Patient 3: after treatment for Cushing’s disease.

**Table 3:** In silico tools used to analyze the pathogenicity score of the *LMNA* p.R545H variant.

In Silico Tool	Score	Prediction	Basis Algorithm
Polyphen-2 ^1^	0.999	Probably Damaging	Protein structure/function and evolutionary conservation
Mutation Taster ^2^	29	Disease Causing	Protein structure/function and evolutionary conservation
CADD ^3^	26.2	Very Likely Deleterious	Contrasts annotations of fixed/nearly fixed derived alleles in humans with simulated variants
FATHMM ^4^	−4.90	Damaging	Evolutionary conservation

^1^ Polyphen-2 score: from 0.000 (most probably benign) to 1 (most probably damaging). ^2^ Mutation Taster score: from 0.000 to 215. ^3^ CADD score: >20 means that a variant is amongst the top 1% of deleterious variants in the human genome. ^4^ FATHMM score: ≤1.5 (damaging), ≥1.5 (tolerated). (http://genetics.bwh.harvard.edu/pph2/; http://www.mutationtaster.org/; https://cadd.gs.washington.edu/; http://fathmm.biocompute.org.uk/, all last accessed on 8 March 2021).

## Data Availability

The data presented in this study are available on request from the corresponding author.
